# Addiction Hospital Emergency Department: A Five-Year Performance Review

**DOI:** 10.1192/j.eurpsy.2025.982

**Published:** 2025-08-26

**Authors:** R. Peric, E. Novakovic, O. Sbutega Filipović, M. Radusin Čkautović, D. Raketić

**Affiliations:** 1 Special Hospital for Addiction Disorders; 2 Dr Laza Lazarevic Clinic for Mental Disorders, Belgrade, Serbia

## Abstract

**Introduction:**

Addictive disorders are chronic, highly recurrent conditions that often require long-term treatment and a multidisciplinary approach. Exacerbation of a chronic disorders presents a special challenge and requires a quick response from the medical team. Emergency services implement life-saving interventions on a daily basis.

**Objectives:**

The objective of this study is to point out the importance of the Emergency Service and the provision of adequate medical care to people who need emergency intervention due to the abuse of psychoactive substances.

**Methods:**

This retrospective study was conductet from January 2018 to January 2023 and included 4337 persons. Data was collected from the Special hospital for addiction disorders in Belgrade, Serbia, using a sociodemographic questionnaire and medical documentation. All included participents were users of the Emergency services. Unknown persons were excluded from the research.

**Results:**

Out of a total of 4337 participants, 76% were male with an average age of 37.71 ± 9.94, while 24% were female with an average age of 37.89 ± 11.42. Opiate withdrawal syndrome was the most common reason for presentation in both sexes. However, it should be noted that the incidence of occurrence in men is significantly higher (68%), while in women it is slightly lower (close to 50%). Statistically significantly more women appear due to symptoms of acute alcohol intoxication (p<0.05), as well as intoxication with hypnotics and withdrawal symptoms from the anxiety-depressive spectrum. Comparing the reasons for reporting throughout the monitoring period of 5 years, with the exception of 2022, the trend of reporting did not change significantly. Opiate withdrawal syndrome is the most common, followed by acute alcohol intoxication, polytoxicomania and marijuana use.

**Conclusions:**

The Emergency Service is of great importance because a large number of patients are cared for on an annual basis due to various symptoms of addiction. In addition to the training and readiness of the medical team, joint work and coordination of other emergency service institutions is also needed for the most efficient care.

**Disclosure of Interest:**

None Declared

